# Image Entropy-Based Interface Evaluation Method for Nuclear Power Plants

**DOI:** 10.3390/e25121636

**Published:** 2023-12-08

**Authors:** Wenzhe Tang, Shanguang Chen, Yun Lin, Chengqi Xue

**Affiliations:** 1 School of Mechanical Engineering, Southeast University, Nanjing 211189, China; wenzhe.tang@seu.edu.cn (W.T.); shanguang_chen@126.com (S.C.); 2 School of Design Art and Media, Nanjing University of Science and Technology, Nanjing 210014, China; yunlin@njust.edu.cn

**Keywords:** image entropy, interface, evaluation method, nuclear power plants

## Abstract

The digital interface is crucial for nuclear plant operators, influencing their decision-making significantly. However, evaluations of these interfaces often overlook users’ decision-making performance; lack established standards, typically occurring after the design phase; and are unsuitable for large-scale assessments. Recognizing the vital role of interface information, this paper built on our previous research and proposed a method tailored for nuclear power plant interfaces, utilizing image entropy to evaluate the impact of information on decision-making. A comparative analysis with an experimental evaluation method empirically validated the effectiveness of the proposed method. This research offers a unique decision-making-centric method to interface evaluation, providing a standardized, adaptable framework for various design phases and enabling extensive and rapid evaluations.

## 1. Introduction

As nuclear power plants evolve towards digitalization and integration, digital interfaces have become integral to their operation and management [1,2]. These interfaces, serving as the primary information source, profoundly influence the decision-making of operators. The digital interface comprises local elements such as layout, color, font, as well as global elements like the amount of information [3,4]. Prior research indicates that the amount of information can more comprehensively represent an interface’s information transmission capability compared to local elements, playing a significant role in operators’ decision-making processes [5,6]. Thus, it is crucial to evaluate the amount of information within a digital interface.

In evaluating digital interfaces, it is important to understand both the content of the evaluation and the methods used. For content, current evaluations mainly focus on local elements like color, layout, and navigation [7]. Deng (2022) and Wan (2021) have, respectively, emphasized the importance of color and layout to user experience [8,9]. However, evaluations often neglect the consideration of the amount of information in the interface, and there is a lack of research on evaluating the influence of interface elements on decision-making [10,11]. For methodology, evaluations typically adopt one of two approaches: subjective questionnaires and experimental tests. Subjective questionnaires are widely used due to their convenience, facilitating rapid collection of user feedback. Allah et al. (2021) highlighted their effectiveness in revealing user preferences and needs during interface navigation, providing a strong foundation for improving interface design [12]. Experimental tests, while more complex, allow for the observation of user interactions in controlled environments. Chu and Liu (2023) elucidated that such evaluations of user interfaces yield profound insights into user needs and interaction outcomes in human–machine collaboration [13]. However, a general lack of standardized evaluation criteria in current methods may lead to inconsistent findings. Moreover, both subjective and experimental approaches are typically only conducted after the design is completed, which restricts their applicability in fast, large-scale assessments [14,15].

In the context of nuclear power plant interfaces, evaluations are often conducted during the design phase, dealing with numerous interfaces of a specific type [16,17,18]. To address this, we proposed a method to accommodate these rapid and large-scale requirements, specifically designed to address user decision-making challenges in interface evaluation. This method builds on findings from our previous work, which determined that there are upper and lower thresholds to the amount of interface information conducive to decision-making [19]. Leveraging this insight, we used image entropy as an indicator and established its threshold range through a questionnaire-based threshold determination approach. Once these threshold ranges are established for specific categories of interfaces, they can be reapplied in subsequent evaluations.

By conducting a systematic study of this method, we offer additional options for evaluating nuclear power plant interfaces. This method stands out by offering a unified standard, allowing for implementation at any design stage, and facilitating data reuse for batch evaluations. It prioritizes human decision-making needs, leading to a more human-centric, targeted, and practical interface evaluation.

## 2. Interface Information Measurement Model

Interface information refers to the content and data that an interface presents to users, encompassing a variety of elements such as text, graphics, animations, videos, audio, and other multimedia components [20,21,22]. The prominence of specific types of information depends on the field of application. In the operating environment of a nuclear power plant, the interface information concentrates on visual inputs closely related to the plant’s status [23,24], predominantly taking the form of text, numerical values, and graphics [25,26]. To facilitate a more effective evaluation of the interface, it is crucial to convert this information into a format that is quantitatively measurable.

### 2.1. Measuring the Amount of Information

Building on this need for quantification, a variety of measurement techniques, including element counting, interface density, and image entropy, are available [27,28,29].

The element counting technique quantifies information by tallying the number of various visible elements such as text, buttons, and icons [30]. Although a large number of elements often indicates a high amount of information, this technique tends to overlook the significant influence of element organization and layout. These factors are vital for a user’s interpretation of interface content, as they substantially contribute to the perceived amount of information.

The interface density technique accounts for both the number of elements and their organization and layout. It calculates the number of elements per unit area, giving insight into the concentration of interface elements and thus evaluating the amount of information [31]. Despite its advantages, this technique has notable drawbacks; it fails to adequately consider how design alterations and differences among interface elements can impact the user’s perception of the amount of information.

Contrary to the interface density technique, the image entropy technique accounts for design alterations and element differences, drawing upon the concept of entropy from information theory [32]. This technique measures the quantity, complexity, or randomness of image information by evaluating the entropy of pixel intensity distributions, serving as a robust indicator of the information content in an image or graphical user interface [33]. Typically, higher values of image entropy correlate with increased visual complexity and a greater amount of information.

While image entropy proves to be a valuable tool, it also has limitations in measuring the amount of information in an interface, such as its inability to account for the spatial arrangement of elements. Nevertheless, it is particularly well-suited for applications in nuclear power plant interfaces. These interfaces adhere strictly to rigorous design standards, dictating the size, shape, color, and layout of elements [34], which minimizes randomness and ensures a consistent distribution of pixel intensity. These characteristics enhance the applicability of image entropy as an evaluative tool for such contexts. Furthermore, these digital interfaces are rich in graphical components, such as charts and images, which exhibit significant variations in pixel intensity—a scenario that plays to the strengths of image entropy [35]. Additionally, the constant stream of dynamic, real-time updates, ranging from equipment statuses to alerts [36], results in shifts in pixel intensity that image entropy is well-equipped to quantify. Consequently, image entropy emerges as a proficient metric for assessing the amount of information in the interfaces of nuclear power plants.

### 2.2. Image Entropy-Based Measurement Model

The image entropy-based measurement model is designed to effectively quantify the amount of information in an interface. The model is versatile, accepting interfaces in various formats such as JPEG, PNG, and BMP as input. It is important that the interface is unedited and complete to ensure accurate analysis. Upon processing, the model outputs a value indicative of the interface’s image entropy, which directly correlates with the information complexity present. A higher value suggests a more information-dense interface. Below, the specific steps of the model are outlined.

#### 2.2.1. Grayscale Conversion

In color image entropy calculation, there are three primary methods: channel-wise calculation, conversion to grayscale, and joint entropy. The “channel-wise calculation” method processes each color channel (red, green, blue) separately and combines the results, providing a detailed color distribution but at a high computational cost. The “conversion to grayscale” method simplifies the process by converting the image to grayscale before entropy calculation, effective for limited-color applications and giving a general overview of brightness. The “joint entropy” method, the most complex, analyzes all color channel combinations for a comprehensive content assessment but requires extensive data processing. For nuclear power plant interfaces, which typically feature no more than five colors and maintain clear contrast in grayscale, the “conversion to grayscale” method is most suitable. It preserves essential information while reducing computational load.

The initial step of the model involves converting a color image into grayscale. Grayscale conversion is executed by transforming the red, green, and blue (RGB) components of each pixel in the color image into a single grayscale value. Given the human eye’s varying sensitivity to different colors, most sensitivity to green, followed by red, and least to blue, different weights are assigned to each color component. According to the ITU-R BT.601 standard (also known as Rec. 601) [37], the weights are distributed as follows: green at 0.587, red at 0.299, and blue at 0.114. The conversion formula is expressed as:

Y=0.299×R+0.587×G+0.114×B

where Y represents the grayscale value, and R, G, and B correspond to the red, green, and blue components, respectively. This formula utilizes a weighting mechanism that aligns with the human eye’s differential sensitivity to colors, ensuring a perceptually uniform grayscale representation.

#### 2.2.2. Calculation of Grayscale Probability

The second step of the model involves tallying the occurrences of each grayscale level, typically ranging from 0 to 255, within the image. To calculate the probability associated with each grayscale level, these counts are subsequently divided by the total number of pixels in the image. The formula for this calculation is expressed as:

$$p_{i}=\frac{n_{i}}{N}$$

where $p_{i}$ represents the probability of the *i*-th grayscale level, $n_{i}$ denotes the number of pixels at that specific grayscale level, and N is the total pixel count in the image.

#### 2.2.3. Entropy Calculation

The final step of the model is the calculation of entropy. For each grayscale level, the product of the corresponding probability and the logarithm (base 2) of its probability is calculated. This product quantifies the average number of bits needed to encode the information in binary code. The sum of these products is then calculated. Given that the logarithm of probabilities ranging between 0 and 1 yields a negative value, the negative of this sum is taken to ensure that the entropy value remains non-negative, accurately representing the expected amount of information. The entropy formula is expressed as:

$$H=-\sum_{i=1}^{L}p_{i}\log_{2}p_{i}$$

where H represents the image entropy, $p_{i}$ denotes the probability of a particular grayscale level across the image, and L corresponds to the cumulative count of grayscale levels present in the image.

This model draws upon the principles of Shannon entropy, a pivotal concept in information theory measuring the amount of information. In image processing, entropy is utilized to evaluate an image’s complexity, texture features, and overall information content. The resulting entropy value is a direct indicator of the amount of information. A higher entropy signifies a greater amount of information, while a lower entropy indicates a more uniform and consistent image.

## 3. Evaluation Method

The image entropy-based measurement model serves as a robust metric due to its capability to quantify the amount of information in an interface. In this section, a method for interface evaluation is proposed, elucidating the application of this model to garner insightful and meaningful results.

### 3.1. Evaluation Procedure

**Figure 1:**
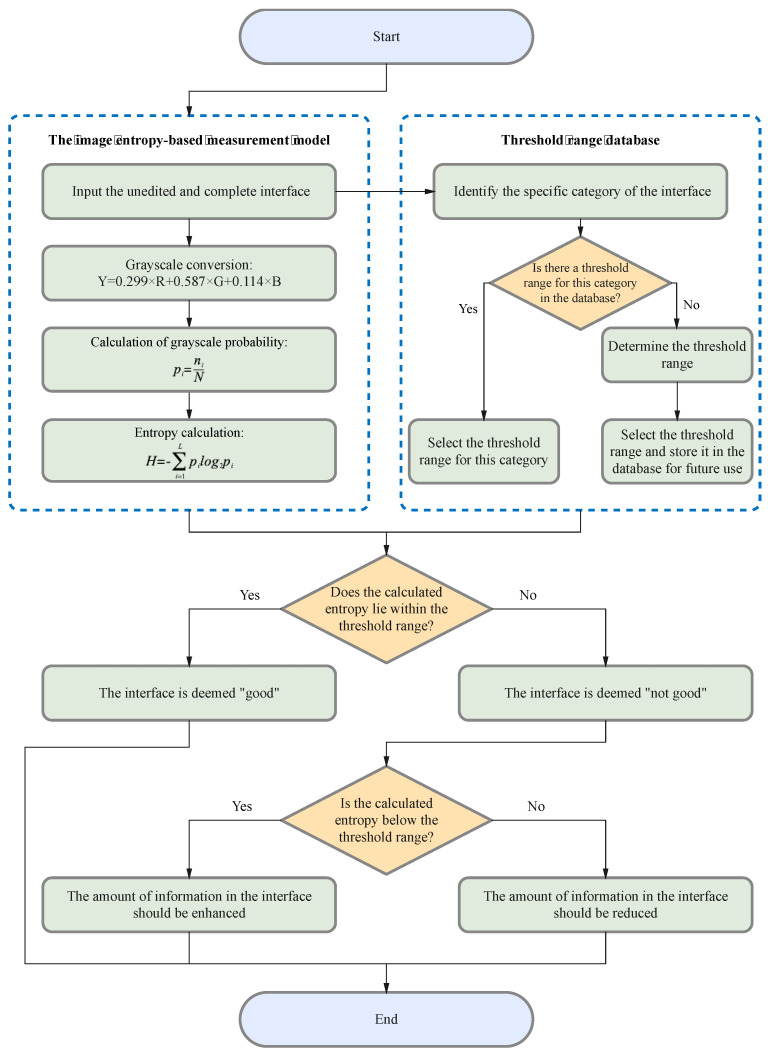
The procedure of the evaluation method.

The procedure of the evaluation method is illustrated in Figure 1. Initially, the image entropy of the interface is calculated using the image entropy-based measurement model. The calculated entropy is then compared to a predefined threshold range, which serves as a benchmark for evaluating its quality. This threshold range corresponds to the category of the interface, with the determination process detailed in Section 3.2 below. Determined threshold ranges for specific categories will be stored in the database for repeated use, facilitating rapid and large-scale evaluation. In the next step, interfaces with entropy values within the threshold range are deemed satisfactory and labeled as “good”. Conversely, those outside of the threshold range are labeled as “not good”, indicating a discrepancy in the amount of information presented.

For interfaces labeled as “not good”, the entropy value provides further insights. A value below the threshold range suggests that the interface could be enhanced by incorporating additional information. In contrast, an entropy value exceeding the threshold range signals a potential information overload, necessitating a reduction in content.

### 3.2. Determination of Threshold Range

The accuracy and stability of the evaluation method hinge crucially on the determination of the threshold range. Given that interfaces within the same category in a nuclear power plant share similar information and task characteristics, and considering the limited number of interface categories, it is feasible to establish specific threshold ranges for each category. To determine these ranges, we employ a “questionnaire-based threshold determination approach”. This approach involves assessing a series of interfaces in the same category, each with distinct image entropies, through a subjective questionnaire. By assigning scores to this series of interfaces, we can deduce the upper and lower thresholds that constitute an appropriate amount of information for that particular category.

The design of the questionnaire in the questionnaire-based threshold determination approach is crucial, as it significantly impacts the final determination of the threshold. Although there is no fixed format for the questionnaire, it should include key parts such as participant information, introduction, reference interfaces, judgment questions, and open-ended questions. The “participant information” part gathers basic details about the participants, including their occupation and previous training experience, to provide context for their responses. The “introduction” part outlines the purpose of the questionnaire and provides guidelines for completing it. The “reference interfaces” part presents interfaces representing the minimum and maximum amounts of information for reference. The “judgment questions” part presents interfaces with varying image entropies in a random order to mitigate order-based bias, and each question corresponds to a specific image entropy. Participants are provided with a series of numerical scores or textual options, such as “minimum” and “excessive”, to rate the amount of information. Finally, the “open-ended questions” part captures more detailed feedback, allowing participants to express their thoughts on the amount of information presented in the interface.

Implementing the questionnaire-based threshold determination approach involves several key steps: participant recruitment, experimental introduction, questionnaire filling, data processing, and conclusion drawing. Throughout the process, the “participant recruitment” step is critical. Potential participants include nuclear power plant operators or graduate students with experience in nuclear power plant projects, as they possess the relevant background knowledge required to provide accurate and reliable data. During the “experimental introduction” step, participants receive essential information about the experiment’s objectives, the tasks they need to complete, and the experiment schedule to ensure they are fully prepared. In the “questionnaire filling” step, participants complete digital or paper questionnaires in a distraction-free environment. Subsequently, the “data processing” step ensures that all collected data are complete and valid. This step also involves converting qualitative answers into quantitative indicators and performing the corresponding statistical calculations. Finally, the “conclusion drawing” step determines the threshold range for a specific interface category based on the processed data results.

### 3.3. Application of the Evaluation Method: An Illustrative Example

**Figure 2:**
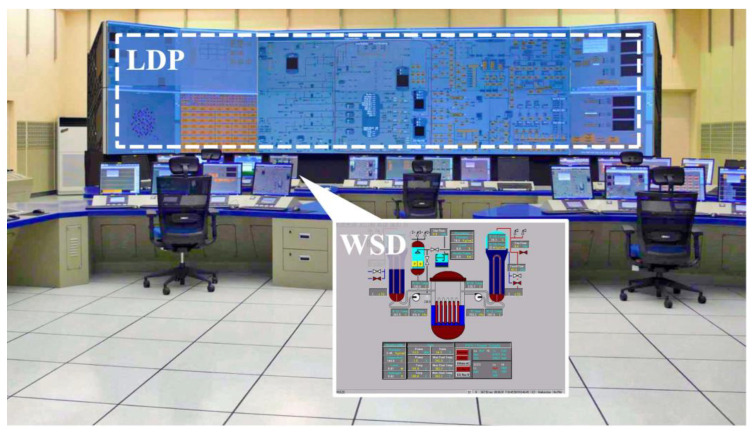
Main control room of the APR1400 nuclear power plant.

Taking the main control room of the APR1400 nuclear power plant as an example, it comprises a large display panel (LDP) and multiple workstation displays (WSD), as shown in Figure 2. The large display panel, primarily utilized by supervisors, facilitates the magnified visualization of the statuses and parameter values of certain plant components. Concurrently, the workstation displays, generally used by operators, provide comprehensive data and information related to plant operations. These displays can be classified into various categories, including alarm displays, system displays, key safety function displays, and computer-based procedure displays. Each display category has different design objectives and necessitates varying amounts of information, so they need to be evaluated individually.

**Figure 3:**
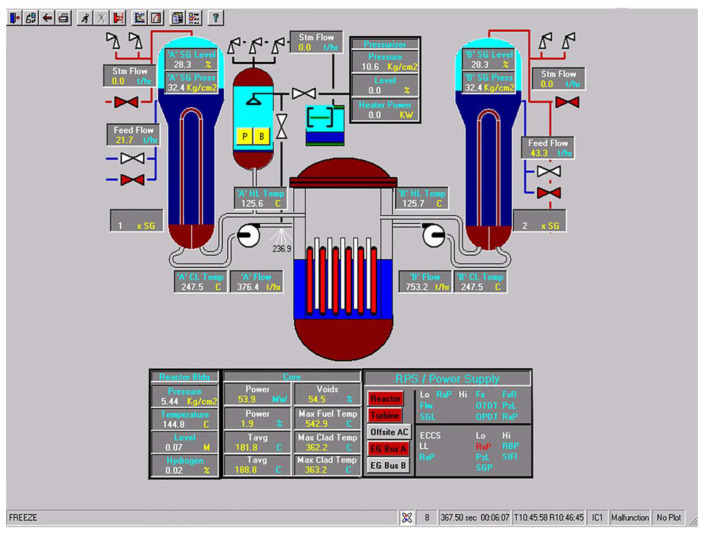
System display interface.

Workstation displays commonly feature a screen resolution of 1024 × 768 pixels. Among the various categories, the system display is prevalent, offering detailed real-time information on specific systems, as depicted in Figure 3. This interface will subsequently serve as an example to illustrate the application of the evaluation method.

#### 3.3.1. Determination of the Threshold Range for the Interface

Since there was no predefined threshold range for this category of interface, the questionnaire-based threshold determination approach was utilized to establish it. The questionnaire applied in this approach is presented in Table 1.

A total of 50 participants were recruited to fill out the questionnaire. These participants were proficient graduate students who had been involved in several nuclear power plant interface development projects; hence, they were viewed as potential specialists in the field. Approximately three days before the study, participants received an informational guide outlining the objectives of the experiment, the assignments they would undertake, and the anticipated timeline. On the day of the experiment, participants engaged with the questionnaire and were asked to complete and submit it within a specific, uninterrupted time frame.

After collecting all 50 questionnaires, the data underwent processing. For each interface, the amount of information was rated using a scoring system that reflected the decision-making utility: minimal and excessive both received 1 point since they are not conducive to effective decision-making, moderate and abundant each received 2 points, while optimal, being the most beneficial for decision-making, received 3 points. Interfaces that achieved a mode of 3 points were considered appropriate for decision-making. Table 2 displays the questionnaire outcomes. Interfaces that garnered a mode of 3 points had an entropy range of 1.28–1.79 (with a 1024 × 768 pixel resolution).

#### 3.3.2. Conducting the Evaluation of the Interface

According to the derived threshold range of 1.28–1.79, when the image entropy of a system display interface lies within this defined range, the amount of information it presents is considered appropriate, striking a balance between being overly simplistic and overly complex. If the image entropy of an interface falls outside of this range, further improvements are required to enhance its usability.

The image entropy of the system display interface depicted in Figure 3 had been calculated to be 1.72 (refer to Section 2.2 for details on the calculation process). Since this value fell within the threshold range of 1.28–1.79, the interface was deemed “good,” indicating that its design was reasonably satisfactory.

## 4. Validation of the Evaluation Method

**Figure 4:**
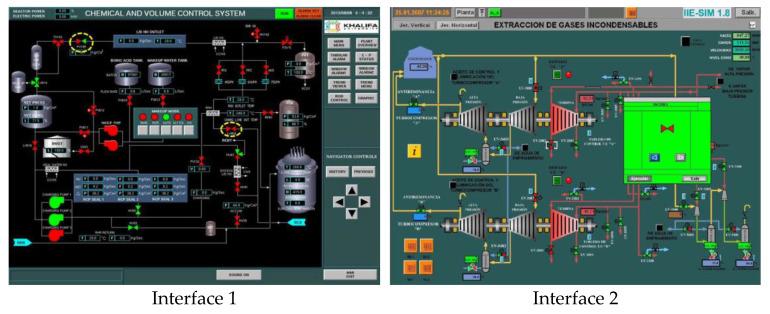

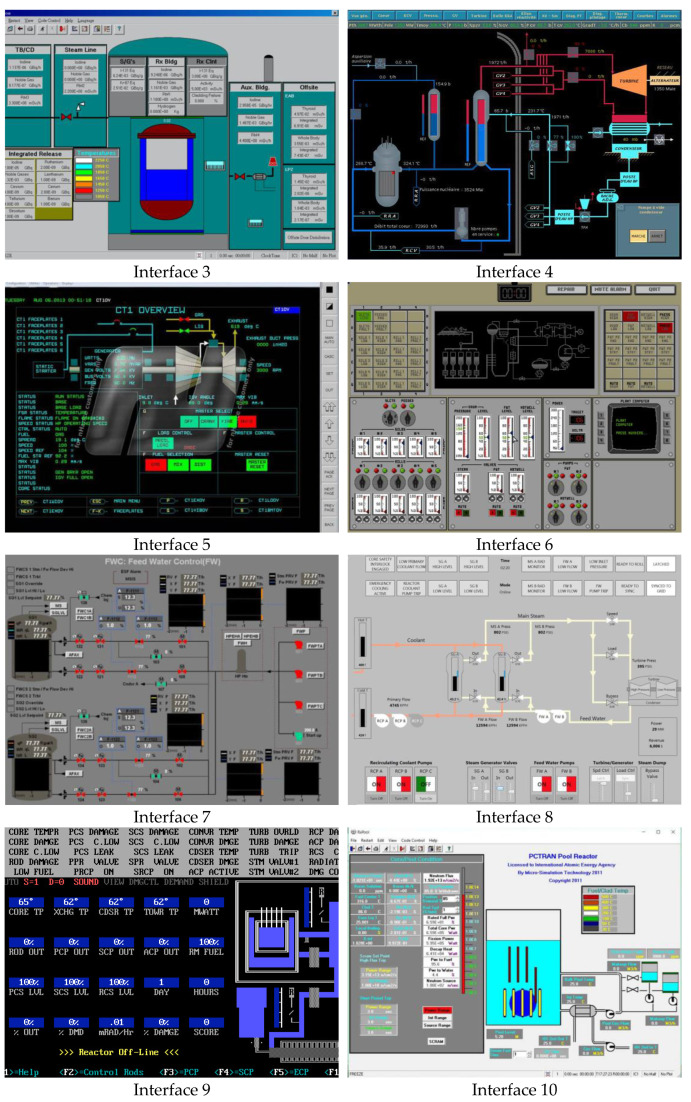

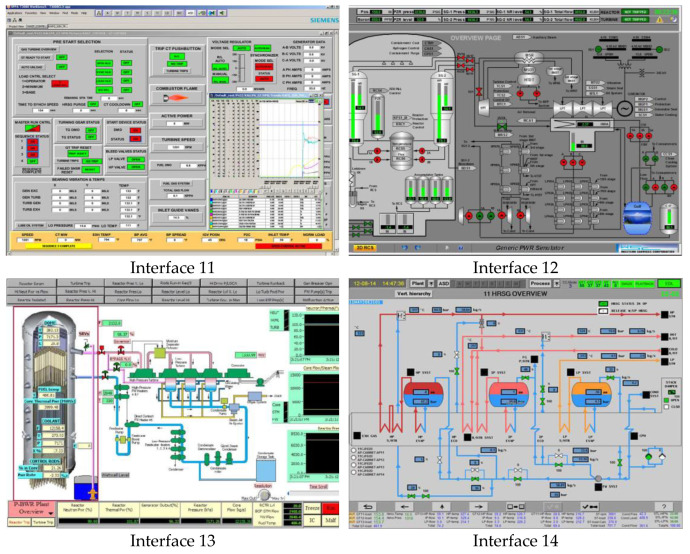
Nuclear power plant interfaces.

To ascertain the efficacy of the proposed evaluation method, a total of 14 interfaces from different nuclear power plants, each boasting a resolution of 1024 × 768 pixels, were meticulously chosen. These interfaces are showcased in Figure 4, encapsulating a broad spectrum of distinct categories. This diverse selection ensures a comprehensive assessment, allowing for a robust validation of the method’s applicability across various interface categories and different nuclear power plants.

### 4.1. Evaluation Using the Proposed Method

The evaluation method detailed in Section 3 was applied to assess the 14 selected interfaces. Specifically, the image entropy for each interface was calculated using the image entropy-based measurement model. Following this, the calculated entropy values were compared against the predefined threshold ranges to gauge the quality of the interfaces. From this comparison, it was determined whether the information content in each interface was optimal or required adjustment.

The application of the proposed evaluation method facilitated assessments of the various interfaces’ quality. The results of this meticulous evaluation are compiled and presented in Table 3, which encompasses the image entropy calculations as well as the assessments relative to the entropy thresholds.

### 4.2. Evaluation Using the Experimental Method

**Figure 5:**
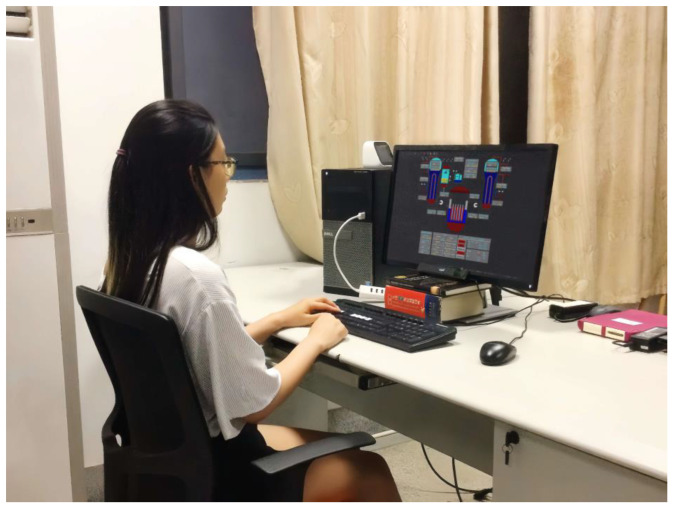
The experimental site.

The interface evaluation was further conducted from an alternative perspective using the conventional experimental method. In this experiment, a within-participant design was adopted, where 30 participants were required to make decisions under the 14 nuclear power plant interfaces. A depiction of the experimental site is provided in Figure 5.

**Figure 6:**
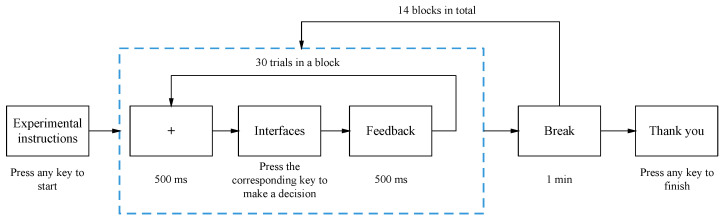
The experimental process.

Participants adhered to the on-screen instructions. Similar to the questionnaire-based threshold determination approach, there were 15 image entropy levels designated for each interface, with each level being presented twice during the experiment. Participants were tasked with completing decision-making tasks for all 14 interfaces, culminating in a total of 420 trials. Each trial commenced with a centered cross shown on the screen for 500 ms, followed by the display of an interface. Participants were then required to rapidly ascertain whether the interface presented any abnormal data and to press the corresponding key to make their decisions. All behavioral data were meticulously logged using E-prime 2.0 software. Figure 6 provides an illustration of the experimental process.

The participants’ decision-making accuracy and reaction time were meticulously recorded. To better assess the impact of the amount of interface information on user decision-making, a new variable named “decision-making efficiency” was defined. It was calculated as the quotient of decision-making accuracy and reaction time, illustrated by the formula:

$$DME=\frac{ACC}{RT}$$

where *DME* represents decision-making efficiency, *ACC* represents decision-making accuracy, and *RT* represents reaction time.

Higher decision-making accuracy and shorter reaction time both contribute to greater decision-making efficiency. This efficiency reflects an overall concept where higher decision-making efficiency indicates that the amount of information presented is more conducive to decision-making. The calculated decision-making efficiency for each of the 14 interfaces, spanning all individual participants, are compiled and presented in Table 4.

Decision-making efficiency can differ among individuals, even when interacting with the same interface. Thus, it is crucial to conduct a correlation analysis on the decision-making results. The Pearson correlation coefficient $r_{xy}$ is computed as:

$$r_{xy}=\frac{n\left(\sum xy\right)-(\sum x)(\sum y)}{\sqrt{\left[n\sum x^{2}-{\left(\sum x\right)}^{2}][n\sum y^{2}-{\left(\sum y\right)}^{2}\right]}}$$

where $r_{xy}$ is the correlation coefficient between X and Y (X and Y correspond to the columns representing two different participants in Table 4). *n* is the number of interfaces. ∑xy is the sum of the products of X and Y. ∑x and ∑y are the sums of X and Y, respectively. $\sum x^{2}$ and $\sum y^{2}$ are the sums of squares of X and Y, respectively.

Given that each participant was assessed independently, a correlation analysis can be conducted between each participant’s quantitative outcomes for the 14 interfaces and those of other participants.

The value of $r_{xy}$ lies in the range $\left[-1,+1\right]$. A positive value, $r_{xy}>0$, indicates a positive correlation, where one variable tends to increase as another variable increases. Conversely, a negative value, $r_{xy}<0$, implies a negative correlation, where one variable tends to decrease as another variable increases. When $r_{xy}=0$, it indicates no linear correlation between the variables. The strength of the correlation is further classified based on the absolute value, where $\left|r_{xy}\right|<0.4$ denotes a weak correlation, $0.4\leq \left|r_{xy}\right|<0.7$ signifies a moderate correlation, and $0.7\leq \left|r_{xy}\right|\leq 1$ indicates a strong correlation.

**Figure 7:**
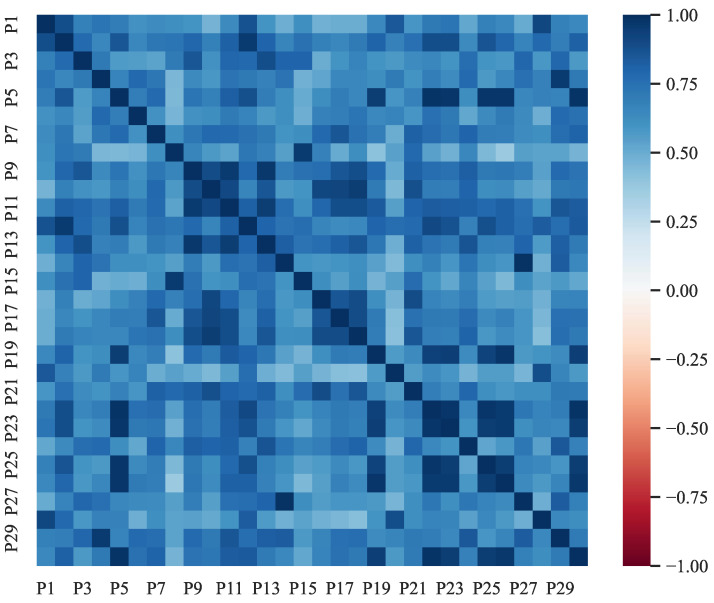
Correlation heatmap for various pairs of participants.

Figure 7 presents the correlation heatmap for various pairs of participants based on decision-making efficiency. Based on the data, it is evident that except for the pair consisting of Participant 8 and Participant 26, whose correlation coefficient was below 0.4, all other pairs had correlation coefficients above 0.4, indicating significant correlation. This result is encouraging, showing that different participants tend to have consistent decision efficiency when interacting with the same interface. It reflects the rationality of the sample selection, the reliability of the experimental design, and the validity of the experimental data.

Normalization and averaging methods were utilized to process the data from Table 4. The formula for normalization is as follows:

$$x’=\frac{x-\min\left(x_{p}\right)}{\max\left(x_{p}\right)-\min\left(x_{p}\right)}$$

where x’ is the normalized value, x is the original value, $\min\left(x_{p}\right)$ and $\max\left(x_{p}\right)$ represent the minimum and maximum values within each participant’s data for the 14 interfaces, respectively. Averaging involved calculating the mean value of the normalized decision-making efficiency for each interface across all 30 participants. Through this process, the decision-making efficiencies for each interface were adjusted to a common scale, facilitating a more straightforward comparison.

To categorize the interfaces based on their decision-making efficiency, the K-means clustering analysis was employed, with the focus solely placed on the “Decision-making efficiency” dimension. With the number of clusters, K, set to 2, the interfaces were aimed to be segregated into two distinct categories: high decision-making efficiency and low decision-making efficiency.

**Figure 8:**
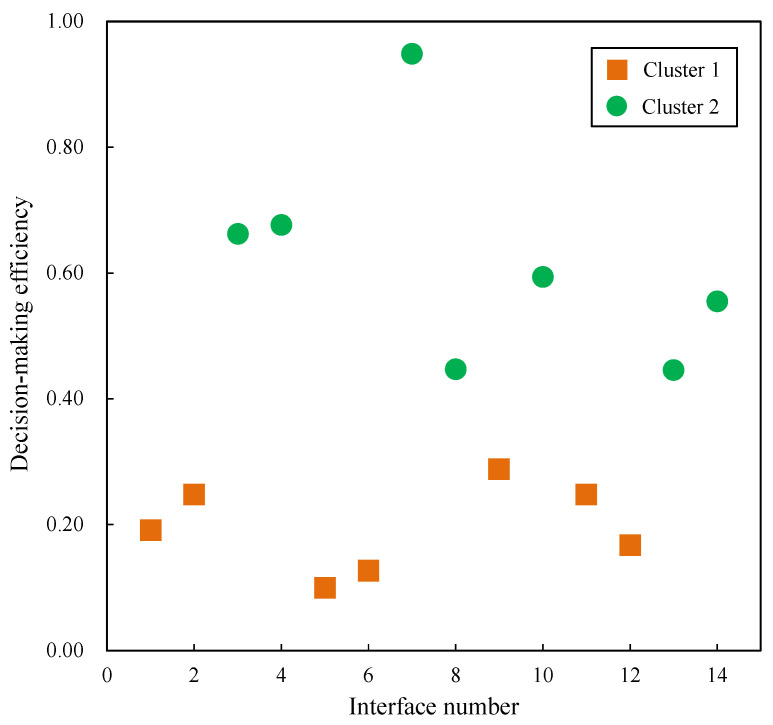
K-means cluster analysis.

The analysis began by randomly selecting two data points to serve as the initial cluster centers. The Euclidean distance was used to assign each data point to the nearest cluster center. The cluster centers were adjusted to minimize the distance from the centers to each data point, and this process was repeated until convergence was achieved. Finally, the first cluster center, representative of the low decision-making efficiency group, showcased a value of 0.20. In contrast, the second cluster center, standing for the high decision-making efficiency group, displayed a value of 0.62. Following this classification, interfaces 1, 2, 5, 6, 9, 11, and 12 were categorized into the first cluster, labeled as “Cluster 1”. Conversely, interfaces 3, 4, 7, 8, 10, 13, and 14 were assigned to the second cluster, labeled as “Cluster 2”. Visual inspection of the clustering results can be achieved through a scatter plot, as shown in Figure 8, where data points are distinctly shaped and colored based on their assigned cluster.

To assess the significance of the clustering, an analysis of variance (ANOVA) was conducted. When *K* = 2, the ANOVA test resulted in *F* (1,12) = 36.30, *p* < 0.001, indicating a significant difference in decision-making efficiency between the two identified clusters.

Interfaces with high decision-making efficiency were evaluated as “Good”, and interfaces with low decision-making efficiency were evaluated as “Not good”. The results of this evaluative process, conducted using the experimental method, are compiled and presented in Table 5.

### 4.3. Comparative Analysis of Results

Upon conducting a comparative analysis of the evaluation results obtained from the proposed method and the experimental method, a high correlation was observed between the outcomes of the two methodologies, as detailed in Table 6.

Interface No. 13 emerged as the sole discrepancy. As observed in Table 3, the image entropy of Interface No. 13 was 6.68, which slightly exceeds the threshold range of 4.25–6.61. Consequently, it is very close to being considered a good interface, which could potentially explain the evaluation bias. Generally, all other interfaces demonstrated consistent results across both methods, achieving a match rate of 92.86%. This high level of consistency reflects the effectiveness of the proposed method.

The comparison results highlight two key points. Firstly, there is a close relationship between the amount of information in an interface and decision-making efficiency. The threshold range derived from the questionnaire, which was based on the amount of information, aligns well with the decision-making performance results obtained through the experimental method. This consistency validates the use of image entropy as a reliable metric for evaluating interface quality. Secondly, the proposed evaluation method proves to be accurate and stable. It demonstrates a strong capability to accurately assess the quality of interfaces within a specific category, and it is applicable across various interface categories. Once the threshold range has been established, it can be repeatedly utilized to quickly evaluate similar interfaces within the same category. This method offers convenience for interface designers in controlling information during the design process and for evaluators in conducting batch and efficient evaluations of interfaces.

## 5. Conclusions

The primary objective of this study was to introduce an evaluation method tailored for nuclear power plant interfaces, building upon our previous research which highlighted the existence of a beneficial range of interface information for decision-making. This paper delves into the evaluation method, encompassing the selection of information measurement techniques, outlining the evaluation procedure, determining the evaluation threshold ranges, and validating the method through the experimental method.

Different from existing evaluation methods for nuclear power plant interfaces, such as subjective methods (questionnaires, heuristic evaluations) and objective methods (Hick’s Law, simulation experiments), this new method provides a unique perspective on interface evaluation. It evaluates interfaces based on whether the information presented supports effective user decision-making, particularly oriented towards human decision-making needs, thus deepening the current understanding of interface evaluation. Furthermore, the method’s basis on image entropy and threshold range judgment endows it with characteristics of being rapid, suitable for large-scale applications, and unaffected by the design stage. Unlike questionnaires, which rely on prolonged user feedback and data analysis, this method utilizes image entropy as a flexible and timely evaluation metric. In contrast to heuristic evaluations, which depend heavily on the evaluator’s design and user experience expertise, this method employs objective quantitative metrics, reducing dependency on expert knowledge and making the process more standardized and objective. Compared to Hick’s Law, which primarily focuses on decision time and the number of options, this method is better suited for complex scenarios in nuclear power plants, offering a more comprehensive understanding of the interface’s impact on decision-making. Additionally, this method outperforms simulation experiments in speed and cost-effectiveness. While simulations require complex setups and lengthy processes, this method can be rapidly applied at any design stage without the need for expensive equipment or intricate setups, significantly reducing evaluation costs and time.

A limitation of this method is the necessity to predefine a threshold range when no existing range is available. This threshold range is dependent on the specific category of the interface. However, determining the threshold range for nuclear power plant interfaces is relatively straightforward, given that each category has its own specific design guidelines and tasks. Future studies should focus on optimizing the process of determining the threshold range. Additionally, combining existing heuristic evaluations, experimental evaluations, and other evaluation methods could lead to a comprehensive evaluation system. Such a system would facilitate thorough evaluations of nuclear power plant interfaces. 

## Figures and Tables

**Table 1:** Questionnaire for determining the information threshold range for the system display interface.

Part 1. Participant information 1. Your name: 2. Your job: (Operator, Supervisor, Technician, etc.) 3. Your training experience: (Please describe any relevant training you’ve had related to nuclear power plant operations)		
Part 2. Introduction This questionnaire aims to collect feedback on how varying amounts of information in nuclear power plant system display interfaces influence decision-making. Your insights will guide us in determining the ideal information threshold range for these interfaces. You will see various interface images, each representing one of five information levels: minimal, moderate, optimal, abundant, or excessive. Please identify which level each image corresponds to, particularly in terms of its informativeness for your decision-making. For your reference, examples of interfaces with the minimum and maximum amounts of information have been provided.		
Part 3. Reference interfaces		
Interface with the minimum amount of information		Interface with the maximum amount of information
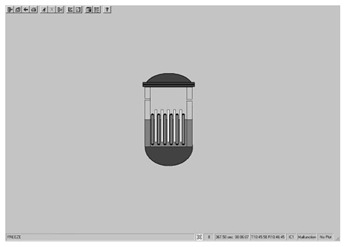		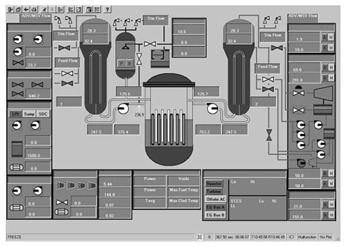
Part 4. Judgment questions		
No. 1	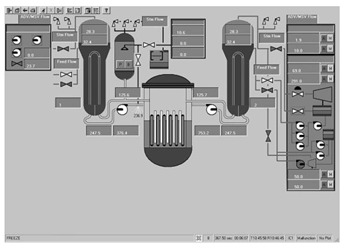	Level of the amount of information: o Minimal o Moderate o Optimal o Abundant o Excessive
No. 2	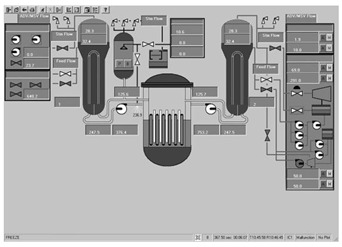	Level of the amount of information: o Minimal o Moderate o Optimal o Abundant o Excessive
No. 3	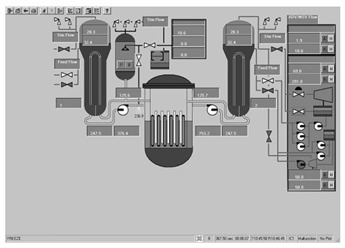	Level of the amount of information: o Minimal o Moderate o Optimal o Abundant o Excessive
No. 4	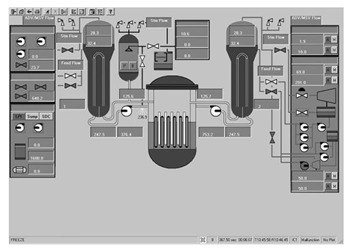	Level of the amount of information: o Minimal o Moderate o Optimal o Abundant o Excessive
No. 5	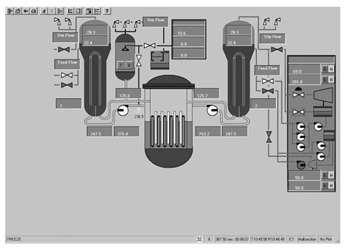	Level of the amount of information: o Minimal o Moderate o Optimal o Abundant o Excessive
No. 6	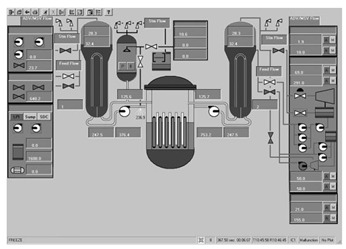	Level of the amount of information: o Minimal o Moderate o Optimal o Abundant o Excessive
No. 7	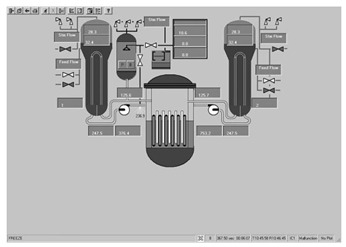	Level of the amount of information: o Minimal o Moderate o Optimal o Abundant o Excessive
No. 8	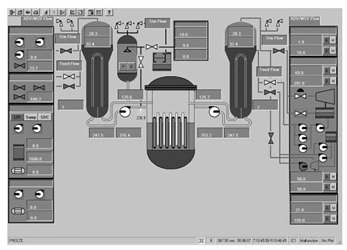	Level of the amount of information: o Minimal o Moderate o Optimal o Abundant o Excessive
No. 9	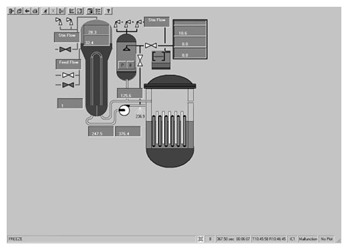	Level of the amount of information: o Minimal o Moderate o Optimal o Abundant o Excessive
No. 10	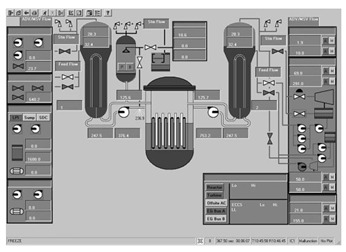	Level of the amount of information: o Minimal o Moderate o Optimal o Abundant o Excessive
No. 11	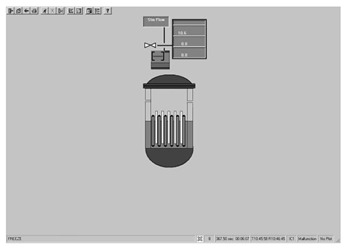	Level of the amount of information: o Minimal o Moderate o Optimal o Abundant o Excessive
No. 12	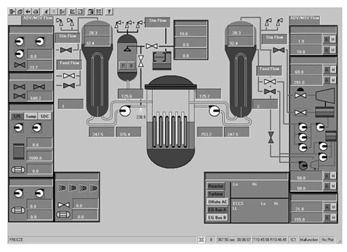	Level of the amount of information: o Minimal o Moderate o Optimal o Abundant o Excessive
No. 13	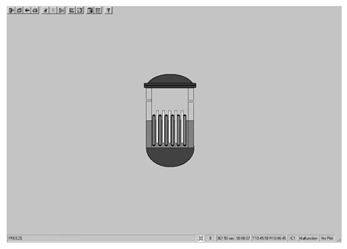	Level of the amount of information: o Minimal o Moderate o Optimal o Abundant o Excessive
No. 14	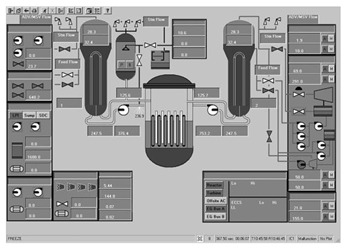	Level of the amount of information: o Minimal o Moderate o Optimal o Abundant o Excessive
No. 15	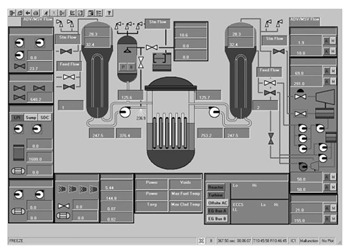	Level of the amount of information: o Minimal o Moderate o Optimal o Abundant o Excessive
Part 5. Open-ended questions 1. In your experience, what aspects contribute most to the amount of information in the interface? 2. How does the amount of information in the interface affect your decision-making? 3. What are the features of an ideal nuclear power plant interface in your view?		

**Table 2:** Questionnaire outcomes.

Judgment Questions	Image Entropy	Mode
No. 1	1.79	3
No. 2	1.84	2
No. 3	1.70	3
No. 4	1.95	2
No. 5	1.63	3
No. 6	2.00	2
No. 7	1.28	3
No. 8	2.05	2
No. 9	1.18	2
No. 10	2.15	1
No. 11	0.56	1
No. 12	2.20	1
No. 13	0.37	1
No. 14	2.23	1
No. 15	2.27	1

**Table 3:** Results of image entropy calculation and entropy-threshold assessment.

Interface Number	Image Entropy	Threshold Range	Assessment
1	4.69	3.01–4.29	Not good
2	5.02	3.22–4.25	Not good
3	5.96	3.65–6.12	Good
4	4.28	3.01–4.52	Good
5	5.21	3.02–4.83	Not good
6	5.93	2.51–4.52	Not good
7	4.66	3.11–4.81	Good
8	3.70	3.21–4.05	Good
9	2.35	1.12–1.82	Not good
10	5.75	3.83–5.81	Good
11	6.54	3.54–5.25	Not good
12	4.61	3.23–4.06	Not good
13	6.68	4.25–6.61	Not good
14	5.53	4.24–6.22	Good

**Table 4:** Decision-making efficiency for the 14 interfaces (Data for 6 participants shown; see Appendix A for all 30).

Interface Number	P1	P2	P3	P4	P5	P6
1	0.12	0.18	0.13	0.14	0.17	0.20
2	0.13	0.17	0.14	0.17	0.18	0.16
3	0.14	0.23	0.26	0.28	0.27	0.28
4	0.38	0.32	0.27	0.32	0.25	0.30
5	0.16	0.14	0.19	0.12	0.09	0.17
6	0.13	0.13	0.13	0.13	0.16	0.16
7	0.39	0.48	0.36	0.24	0.38	0.23
8	0.18	0.19	0.20	0.22	0.23	0.18
9	0.19	0.18	0.08	0.15	0.19	0.18
10	0.37	0.34	0.18	0.27	0.35	0.25
11	0.19	0.20	0.18	0.19	0.19	0.19
12	0.12	0.18	0.08	0.09	0.13	0.18
13	0.27	0.27	0.28	0.23	0.15	0.20
14	0.22	0.29	0.20	0.19	0.30	0.27

**Table 5:** Results of decision-making efficiency calculation and cluster-based assessment.

Interface Number	Decision-Making Efficiency	Cluster Analysis	Assessment
1	0.19	Cluster 1	Not good
2	0.25	Cluster 1	Not good
3	0.66	Cluster 2	Good
4	0.68	Cluster 2	Good
5	0.10	Cluster 1	Not good
6	0.13	Cluster 1	Not good
7	0.95	Cluster 2	Good
8	0.45	Cluster 2	Good
9	0.29	Cluster 1	Not good
10	0.59	Cluster 2	Good
11	0.25	Cluster 1	Not good
12	0.17	Cluster 1	Not good
13	0.45	Cluster 2	Good
14	0.56	Cluster 2	Good

**Table 6:** Comparison between the proposed method and the experimental method.

Interface Number	Image Entropy Threshold Range	Decision-Making Efficiency	Entropy-Based Assessment	Cluster-Based Assessment
1	3.01–4.29	0.19	Not good	Not good
2	3.22–4.25	0.25	Not good	Not good
3	3.65–6.12	0.66	Good	Good
4	3.01–4.52	0.68	Good	Good
5	3.02–4.83	0.10	Not good	Not good
6	2.51–4.52	0.13	Not good	Not good
7	3.11–4.81	0.95	Good	Good
8	3.21–4.05	0.45	Good	Good
9	1.12–1.82	0.29	Not good	Not good
10	3.83–5.81	0.59	Good	Good
11	3.54–5.25	0.25	Not good	Not good
12	3.23–4.06	0.17	Not good	Not good
13	4.25–6.61	0.45	Not good	Good
14	4.24–6.22	0.56	Good	Good

## Data Availability

Data are contained within the article.

## Appendix A

**Table A1:** Complete decision-making efficiency data for the 14 interfaces (all 30 participants).

**Interface Number**	**P1**	**P2**	**P3**	**P4**	**P5**	**P6**
1	0.12	0.18	0.13	0.14	0.17	0.20
2	0.13	0.17	0.14	0.17	0.18	0.16
3	0.14	0.23	0.26	0.28	0.27	0.28
4	0.38	0.32	0.27	0.32	0.25	0.30
5	0.16	0.14	0.19	0.12	0.09	0.17
6	0.13	0.13	0.13	0.13	0.16	0.16
7	0.39	0.48	0.36	0.24	0.38	0.23
8	0.18	0.19	0.20	0.22	0.23	0.18
9	0.19	0.18	0.08	0.15	0.19	0.18
10	0.37	0.34	0.18	0.27	0.35	0.25
11	0.19	0.20	0.18	0.19	0.19	0.19
12	0.12	0.18	0.08	0.09	0.13	0.18
13	0.27	0.27	0.28	0.23	0.15	0.20
14	0.22	0.29	0.20	0.19	0.30	0.27
**Interface Number**	**P7**	**P8**	**P9**	**P10**	**P11**	**P12**
1	0.16	0.18	0.14	0.14	0.19	0.22
2	0.21	0.25	0.13	0.12	0.16	0.23
3	0.29	0.26	0.30	0.33	0.37	0.28
4	0.29	0.40	0.23	0.22	0.29	0.40
5	0.07	0.18	0.15	0.11	0.15	0.18
6	0.21	0.18	0.15	0.11	0.14	0.15
7	0.33	0.48	0.39	0.34	0.43	0.55
8	0.23	0.14	0.26	0.25	0.33	0.26
9	0.24	0.25	0.17	0.25	0.19	0.21
10	0.27	0.14	0.20	0.20	0.31	0.39
11	0.15	0.19	0.15	0.15	0.17	0.19
12	0.18	0.20	0.15	0.19	0.18	0.15
13	0.24	0.31	0.27	0.25	0.29	0.25
14	0.22	0.27	0.28	0.26	0.32	0.30
**Interface Number**	**P13**	**P14**	**P15**	**P16**	**P17**	**P18**
1	0.16	0.23	0.23	0.18	0.21	0.26
2	0.19	0.27	0.24	0.19	0.23	0.23
3	0.33	0.37	0.32	0.32	0.43	0.43
4	0.25	0.31	0.40	0.29	0.33	0.35
5	0.17	0.20	0.24	0.13	0.20	0.19
6	0.12	0.13	0.27	0.13	0.27	0.20
7	0.42	0.37	0.52	0.34	0.39	0.42
8	0.28	0.25	0.20	0.23	0.32	0.32
9	0.16	0.12	0.25	0.26	0.30	0.31
10	0.22	0.27	0.19	0.22	0.33	0.29
11	0.16	0.21	0.21	0.16	0.22	0.27
12	0.17	0.19	0.20	0.27	0.30	0.29
13	0.30	0.25	0.32	0.24	0.34	0.38
14	0.26	0.21	0.29	0.27	0.34	0.40
**Interface Number**	**P19**	**P20**	**P21**	**P22**	**P23**	**P24**
1	0.15	0.20	0.20	0.19	0.16	0.13
2	0.18	0.23	0.18	0.22	0.19	0.25
3	0.27	0.30	0.27	0.35	0.26	0.34
4	0.25	0.54	0.29	0.33	0.24	0.28
5	0.12	0.35	0.10	0.12	0.13	0.13
6	0.18	0.21	0.12	0.18	0.15	0.15
7	0.38	0.44	0.38	0.48	0.37	0.35
8	0.29	0.33	0.24	0.24	0.20	0.27
9	0.18	0.34	0.27	0.20	0.18	0.22
10	0.32	0.41	0.18	0.40	0.35	0.20
11	0.22	0.28	0.16	0.23	0.18	0.20
12	0.17	0.22	0.18	0.16	0.15	0.12
13	0.18	0.26	0.23	0.18	0.19	0.30
14	0.35	0.33	0.24	0.37	0.33	0.23
**Interface Number**	**P25**	**P26**	**P27**	**P28**	**P29**	**P30**
1	0.23	0.20	0.25	0.20	0.23	0.22
2	0.23	0.23	0.28	0.25	0.26	0.25
3	0.32	0.35	0.39	0.26	0.48	0.39
4	0.29	0.32	0.33	0.39	0.50	0.34
5	0.17	0.13	0.20	0.28	0.19	0.11
6	0.24	0.22	0.17	0.20	0.16	0.22
7	0.48	0.47	0.39	0.43	0.43	0.47
8	0.27	0.33	0.27	0.30	0.39	0.30
9	0.21	0.18	0.12	0.33	0.22	0.25
10	0.41	0.45	0.29	0.40	0.39	0.42
11	0.25	0.23	0.22	0.27	0.23	0.27
12	0.20	0.15	0.19	0.22	0.15	0.18
13	0.19	0.18	0.25	0.28	0.40	0.21
14	0.35	0.45	0.22	0.24	0.30	0.39
